# Co-regulatory effects of hormone and mRNA–miRNA module on flower bud formation of *Camellia oleifera*

**DOI:** 10.3389/fpls.2023.1109603

**Published:** 2023-03-17

**Authors:** Wei Du, Jian Ding, Jingbin Li, He Li, Chengjiang Ruan

**Affiliations:** Institute of Plant Resources, Key Laboratory of Biotechnology and Bioresources Utilization, Ministry of Education, Dalian Minzu University, Dalian, China

**Keywords:** *Camellia oleifera*, flower bud formation, hormone signal transduction, circadian rhythm, transcriptomic, alternate bearing

## Abstract

Few flower buds in a high-yield year are the main factors restricting the yield of *Camellia oleifera* in the next year. However, there are no relevant reports on the regulation mechanism of flower bud formation. In this study, hormones, mRNAs, and miRNAs were tested during flower bud formation in MY3 (“Min Yu 3,” with stable yield in different years) and QY2 (“Qian Yu 2,” with less flower bud formation in a high-yield year) cultivars. The results showed that except for IAA, the hormone contents of GA_3_, ABA, tZ, JA, and SA in the buds were higher than those in the fruit, and the contents of all hormones in the buds were higher than those in the adjacent tissues. This excluded the effect of hormones produced from the fruit on flower bud formation. The difference in hormones showed that 21–30 April was the critical period for flower bud formation in *C. oleifera*; the JA content in MY3 was higher than that in QY2, but a lower concentration of GA_3_ contributed to the formation of the *C. oleifera* flower bud. JA and GA_3_ might have different effects on flower bud formation. Comprehensive analysis of the RNA-seq data showed that differentially expressed genes were notably enriched in hormone signal transduction and the circadian system. Flower bud formation in MY3 was induced through the plant hormone receptor TIR1 (transport inhibitor response 1) of the IAA signaling pathway, the miR535-*GID1c* module of the GA signaling pathway, and the miR395-JAZ module of the JA signaling pathway. In addition, the expression of core clock components *GI* (*GIGANTEA*) and *CO* (*CONSTANS*) in MY3 increased 2.3-fold and 1.8-fold over that in QY2, respectively, indicating that the circadian system also played a role in promoting flower bud formation in MY3. Finally, the hormone signaling pathway and circadian system transmitted flowering signals to the floral meristem characteristic genes *LFY* (LEAFY) and *AP1* (APETALA 1) *via FT* (FLOWERING LOCUS T) and *SOC1* (SUPPRESSOR OF OVEREXPRESSION OF CO 1) to regulate flower bud formation. These data will provide the basis for understanding the mechanism of flower bud alternate formation and formulating high yield regulation measures for *C. oleifera*.

## 1 Introduction

*Camellia oleifera* is one of the four major woody oil tree species in the world. The seed oil, containing up to 75% oleic acid, is an important source of high-quality edible oil (Quan et al., 2022). The widespread phenomenon of alternate bearing in *C. oleifera* often results in a 30%–50% yield reduction. The main reason for alternate bearing is related to the difference in the number of flower buds formed in adjacent years. The alternate-bearing cultivars of *C. oleifera* in the productive year have few flower buds on the branches. This results in less fruit the following year, leading to the alternate fruiting phenomenon (Jia et al., 2018). However, the flowering and fruiting characteristics of *C. oleifera* are different from those of other economically important species. March–June is the period of flower bud formation and fruit growth (Deng et al., 2020). Fruits and flower buds exist simultaneously on the same branch. There are no reports on the mechanism of “alternate flower bud” formation in *C. oleifera*, and the effect of hormones produced by many fruits on flower bud formation in high-yield years of *C. oleifera* remains unclear.

Flower bud formation is induced by many factors, such as photoperiod, temperature, hormones, and age-related signals (Lee and Lee, 2010). Based on recent studies on the molecular mechanisms of flower bud formation, the responses of plants to various endogenous and exogenous signals are mediated *via* the circadian rhythm, vernalization, autonomous, age-related, and hormone signal transduction pathways (Thomson and Wellmer, 2019). The mechanism of action of different signaling pathways is essentially the release of the corresponding inhibitory factor. In the circadian rhythm pathway, an increase in sunshine duration can relieve the inhibition of CDF (Dof zinc finger protein DOF) on CO (CONSTANS) and FT (FLOWERING LOCUS T) (Creux and Harmer, 2019). Low-temperature induction and spontaneous pathways in the vernalization and autonomic pathways relieve the inhibitory effect of FLC (FLOWERING LOCUS C) and VRN3 (Recoverin 3) on FT (Distelfeld et al., 2009). Meanwhile, age-related pathways can relieve the repression of SOC1 (SUPPRESSOR OF OVEREXPRESSION OF CO 1) and FLC by AP2 (APETALA 1) through miRNA156 and miR172 (Binenbaum et al., 2018), and the GA pathway can relieve DELLA inhibition on FT, SPLs (photoproduct lyase), and SOC1 by binding GA to GID1 (Bao et al., 2020). The signaling molecules eventually transmit regulatory signals to the downstream *via* the expression of flower meristem characteristic genes (*LFY*, *AP1*, *CAL*, and *TFL*), maintain flower meristem characteristics, and form flower buds (Ó’Maoiléidigh et al., 2014). MicroRNAs (miRNAs), a class of small non-coding RNAs with a length of about 18–24 nucleotides, play an important regulatory role in flower bud formation by inhibiting targeted mRNA (Bartel, 2004). Currently, miRNA families such as miR156, miR172, and miR159/319 have been confirmed to promote or inhibit flower bud formation by targeting receptor proteins or transcription factors in the above pathways, such as the *SPL* family, *AP*2, and *MYB*. In *C. oleifera*, *CoFT* has been successfully isolated, and transcriptome sequencing of flower development showed that FT was a key gene during flower formation (Hu et al., 2014). The expression of *FT* showed diurnal rhythms under both long-day and short-day conditions and was photoperiod-dependent (Lei et al., 2017). Previous studies on *C. oleifera* buds were mostly concerned with sepal, petal, estrogen, and stamen formation after the formation of the flower primordium and investigated the effects of hormones and key genes during the differentiation of flowers (Li et al., 2013; Lu et al., 2021). However, the floral primordium had already formed, and the subsequent differentiation process had limited influence on the number of *C. oleifera* flowers. Few studies had been focused on the end of April to early May after spring shoot germination, which is the key period for the transformation of top or axillary buds into flower buds, and the number of flower buds directly determines the yield of *C. oleifera* in the following year (Chen et al., 2018). Therefore, it is of great theoretical and practical significance to further study the regulatory mechanism of flower bud formation during this critical period to increase the yield of *C. oleifera*.

Advances in *C. oleifera* genomics have revealed novel findings on gene function and provided valuable genomic resources for the genetic improvement of oil crops (Lin et al., 2022). Research progress on bud formation is insufficient compared with that on model plants or other economic forest crops, and there are still no reports focusing on the regulatory mechanism of alternate bud formation in *C. oleifera*. In this study, we determined the key period of *C. oleifera* flower bud formation by observing the structure of flower bud formation. Changes in endogenous hormone levels in different tissues were detected using solid-phase extraction-LC-MS/MS to analyze the effects of endogenous hormones on flower bud formation. The key genes and miRNAs regulating bud formation in *C. oleifera* were explored using high-throughput transcriptome sequencing technology. These data will not only provide new evidence for the functional study of miRNAs and the analysis of the regulatory mechanism of plant flower bud differentiation but also lay the foundation for high-yield management and high-yield cultivar selection of *C. oleifera*.

## 2 Materials and methods

The experimental site is in Tongren City, Guizhou Province, China (E: 108°54’, N: 27°17’). The site where the experiment was performed had a mean annual rainfall of 1180 mm, a mean annual temperature of 16.5°C, mean annual sunshine rate of 1,330, a relative humidity of 80%, and a frost-free period of 299 days. Two cultivars, “Qian Yu 2” (QY2) and “Min Yu 3” (MY3), were selected because they had similar phenotypic characteristics and genetic backgrounds, but a different number of flower buds formed in consecutive years. The genetic similarity coefficient between two cultivars was 0.886 based on ISSR marker analysis. The phenological timing of these two cultivars was basically the same throughout successive growing seasons. In the current year, branches of QY2 (with more fruits and fewer buds, alternate flower bud formation), and MY3 (with stable flower bud formation and yield), which were in the early stage of the differentiation of flower buds, were collected on 7, 14, 21, and 30 April 2022. Meristem tips (terminal buds and axillary buds), stems, fruits, and leaves were collected from high, medium, and low positions. The same tissue was mixed, frozen with liquid nitrogen after collection, and then stored at −80°C. After collection, IAA, GA_3_, ABA, JA, SA, and tZ in different tissues were extracted and determined.

### 2.1 Determination of endogenous hormones in different tissues during bud formation in *C. oleifera*

The samples were ground into powder using liquid nitrogen and then added to 4 ml of extraction solvent (75% methanol and 5% formic acid) for a 24-hour extraction. The extract was centrifuged at 10,000×*g* for 15 min, and then 2 ml of the extract was added to clean the precipitate twice before combining the extract. The extract was concentrated to a constant weight by vacuum centrifugation to remove methanol and re-dissolved in 10 ml of 0.6 mol/L formic acid. Hormone analysis was performed using high-performance liquid chromatography-tandem mass spectrometry (LC-MS/MS) with modifications (Hashiguchi et al., 2021), and endogenous hormones were purified using MCX (Oasis Waters, USA) solid-phase extraction. After injection, the samples were eluted with 2 ml methanol and 3 ml ammoniated methanol (60% methanol v/v, 0.35 mol/L NH_3_·H_2_O). The eluate was combined and concentrated to 10 ml, and the samples were filtered through a 0.45 μm filter membrane before being analyzed using HPLC-MS/MS. HPLC parameters were as follows: in ESI (+) mode, mobile phase A was water with 0.1% formic acid, and mobile phase B was methanol; in ESI (−) mode, mobile phase A was water, and phase B was methanol. Shimadzu InertSustain AQ-C18 column (50 × 2.1 mm; diameter 1.9 μm) was used with an isocratic elution (B: 70%) at a flow rate of 0.2 ml/min for 10 min. IAA and tZ were detected *via* tandem quadrupole mass spectrometry (AB API 3200, USA) in ESI+ to ionize the target ions. ABA, GA_3_, JA, and SA were ionized in ESI−. Other conditions were as follows: ionization voltage, +5,500/−4,500 V; TEM, 550°C; curtain air pressure, 30 psi; scanning mode; and multiple reaction monitoring (MRM) mode. The corresponding MRM parameters for six hormones are shown in **Table S1**. Hormone concentrations were calculated using an external standard method. To explore the distribution of endogenous hormones in different tissues at the early stage of flower bud differentiation and their effects on flower bud differentiation, the mean value and coefficient of variation of each endogenous hormone in the four stages of the two *C. oleifera* cultivars were calculated. A total of three biological replications were performed for each sample. Prism 9 software was used for correlation analysis and mapping.

### 2.2 RNA extraction and sequencing

MY3 and QY2 buds collected on 14, 21, and 30 April were used for RNA extraction. The *C. oleifera* bud RNA samples were extracted using TRIzol reagent (Takara, Japan). RNA samples with three biological replications were tested for purity, concentration, and integrity to ensure they were qualified for transcriptome sequencing. First, 3′- and 5′-SR adaptors were ligated, followed by reverse transcription of the synthetic first chain. PCR amplification and size selection were performed. In order to obtain small RNA libraries. Rubber-cutting recycling was also used to obtain these libraries. Finally, the PCR products were purified (AMPure XP system, Beckman Coulter, USA), and library quality was assessed. In accordance with the manufacturer’s instructions, clustering of the index-coded samples was performed using a cBot Cluster Generation System and the TruSeq PE Cluster Kit v4-cBot-HS. Sequencing of single-end reads was performed on the Illumina platform (Illumina, USA) following library preparation. The raw FASTQ data were first processed using in-house Perl scripts. Reads containing adapters, poly-N, and low-quality reads were removed. Sequences shorter than 18 nt and longer than 30 nt were removed from the reads. A high-quality, clean dataset was used for all downstream analyses. Additionally, the Q20, Q30, and GC contents of the clean data were calculated simultaneously. Randfold tools were used to predict the secondary structure of novel miRNAs. TargetFinder software was used for miRNA target gene prediction. It was used as the main basis for predicting miRNA target genes by seed sequence complementarity, sequence conservation, free energy after pairing, UTR base distribution, and tissue distribution correlation.

### 2.3 Identification and analysis of mRNA sequencing data

Clean, high-quality data were used for all downstream analyses. StringTie was used to assemble the transcriptome based on reads mapped to the reference genome. The clean reads of each sample were aligned with the specified reference genome (http://tpdbtmp.shengxin.ren:81/index.html), and the alignment efficiency ranged from 63.47% to 70.18%. GffCompare was used to annotate assembled transcripts and screen for putative RNAs based on unknown transcripts. The sequenced reads would be compared with the reference genome after splicing. If the matched location exists in the gff file, it would be identified as a known gene. If the gff file of the reference genome did not contain annotation information for this location, it would be identified as a new gene that was only specific to the reference genome. We combined CPC2/CNCI/Pfam approaches to sort putative protein-coding transcripts from putative non-protein-coding RNAs. Putative protein-coding RNAs were filtered using the minimum length and exon number thresholds. Candidates containing more than two exons and transcripts longer than 200 nt were further screened using Pfam/CPC2/CPAT/CNCI, tools that distinguish protein-coding genes from non-coding genes. StringTie (1.3.1) was used to calculate the FPKMs (fragments per kilobase of exon model per million mapped fragments) of genes. Each gene group’s FPKM was computed by adding up its transcript FPKMs.

### 2.4 Functional annotation and differential expression analysis

A variety of databases were used for annotation, including Nr, Pfam, KOG/COG, Swiss-Prot, KEGG, and GO. Differential expression analysis of each sample was performed *via* the DESeq2 R package with three biological replicates. To control false discovery rates, Benjamini and Hochberg’s approach was used to adjust the *p*-values. GO function annotation and KEGG signaling pathway annotation were performed to identify the target genes of differentially expressed miRNAs (Kanehisa and Goto, 2000). The GO and KEGG pathways of the targeted genes were tested with Fisher’s exact hypothesis, and enrichment analyses were conducted separately for each KEGG pathway and GO term. A default threshold of *p ≤*0.05 was set for significant differences in GO and KEGG information for the target genes of differentially expressed miRNAs.

### 2.5 Quantitative real-time PCR

Total RNA was extracted from the buds of *C. oleifera* using the TRIZOL reagent (Takara, Japan) (Du et al., 2022). cDNA was synthesized by reverse transcription, and the cDNA samples were mixed with SYBR Green PCR Real Master Mix (Tiangen, China) and 10 μmol/L of each primer. Applied Biosystems 9700 (ABI, USA) was used to conduct PCR. The program settings were as follows: heating for 15 min at 95°C, followed by 42 cycles of 32 s at 95°C, 30 s at 59°C, and 48 s at 67°C. The Primer Quest online software was used to design the qRT-PCR primers (**Table S2**). The fluorescence signal was collected at the 67°C elongation step of each cycle, and relative quantification was achieved using the 2^−ΔΔCt^ method (Schmittgen and Livak, 2008). *Actin* was selected as the internal standard. Three replications were performed on each sample.

## 3 Results

### 3.1 Analysis of flower buds in successive multiple growing seasons

**Figure 1:**
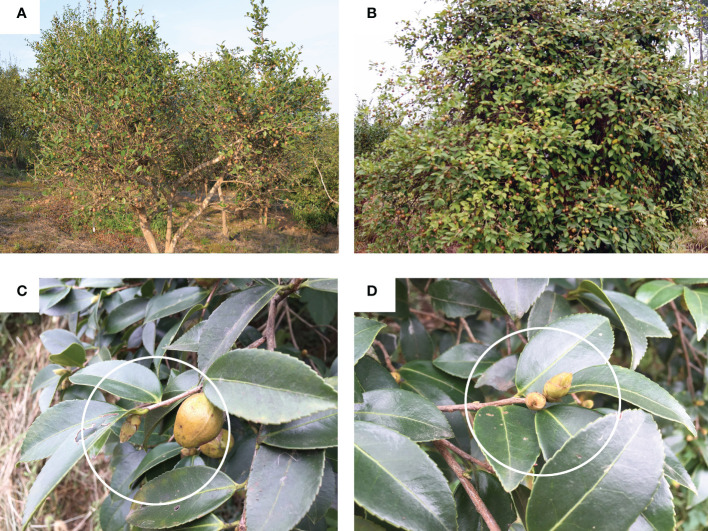
Observation on flower buds and fruits of two *Camellia oleifera* cultivars. **A**: MY3; **B**: QY2; **C**: QY2 in productive year; **D**: QY2 in poor harvest year.

The numbers of flower buds and fruits of MY3 and QY2 are shown in **Figure 1**. MY3 had more fruits in the current year, and a considerable number of flower buds were formed on the branches (**Figure 1A**). The fruit yield of the following year would be equivalent to that of the current year, with no alternate bearing phenomenon in MY3. In QY2, few flower buds were formed (**Figures 1B, C**) in the productive year. There were one to two terminal buds on the branches, with little or no differentiation of the axillary buds, and this resulted in a low number of fruits the following year. The number of flower buds in QY2 was higher in the poor harvest year (**Figure 1D**) than in the productive year, with two to four terminal flower buds and axillary buds easily differentiating into flower buds.

**Figure 2:**
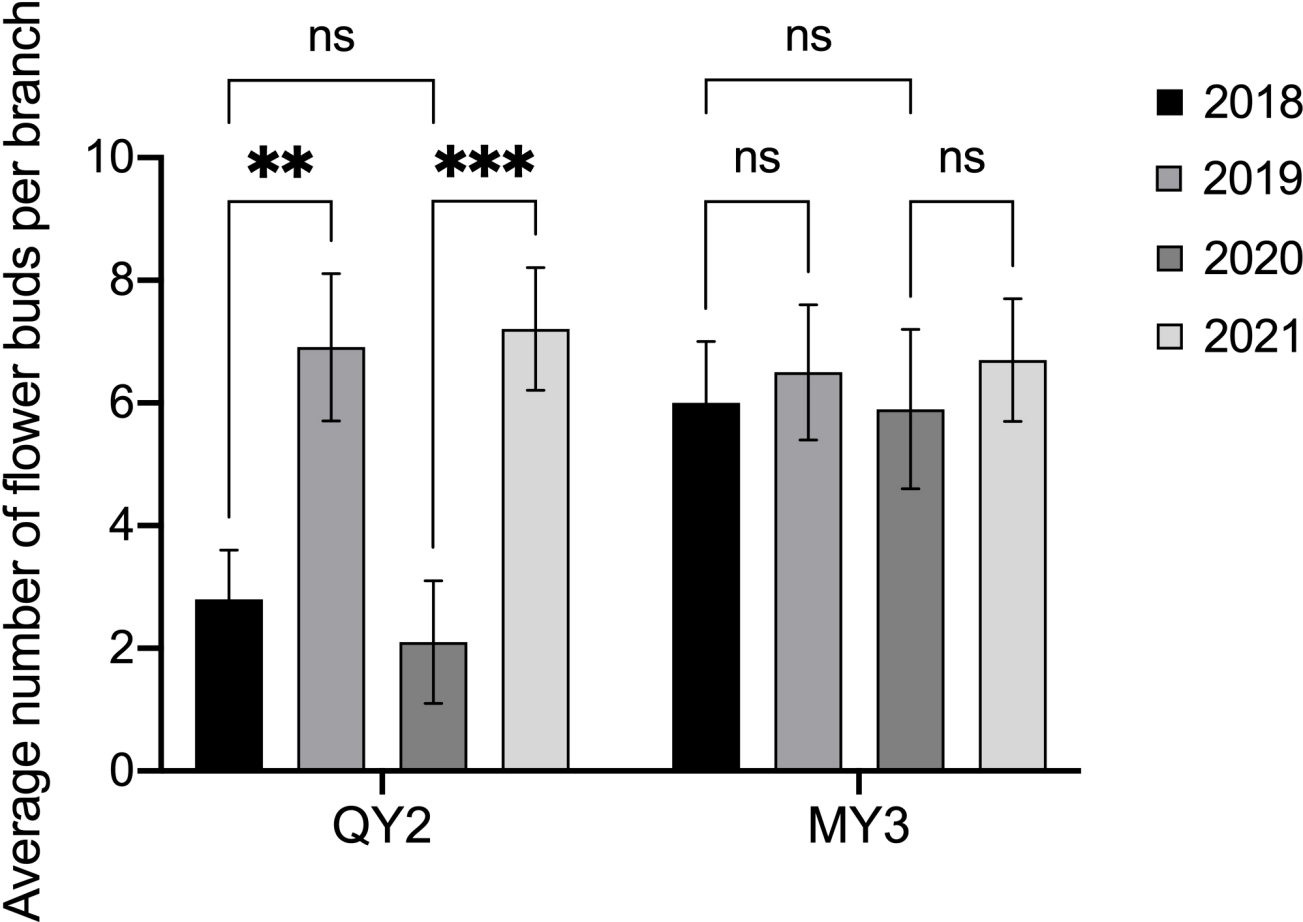
Flower buds in successive multiple growing seasons of two *Camellia oleifera* cultivars. ** p<0.01, *** p<0.001, ns: Not significant. All values represent the means of biological triplicates.

There was a notable difference in the number of flower buds in QY2 that formed in the two consecutive growing seasons (**Figure 2**). There were 3.3 flower buds on average per branch in 2018. The low number of flower buds led to less fruit-bearing and more flower bud differentiation in 2019. The average number of flower buds per branch in 2019 was 7.7, which was considerably higher than that in 2018. The average number of flower buds per branch of MY3 in four consecutive growing seasons from 2018–2021 was approximately 6–7, and there was no notable difference in the number of flower buds differentiated between adjacent or separate growing seasons.

### 3.2 Bud formation process in *C. oleifera*

**Figure 3:**
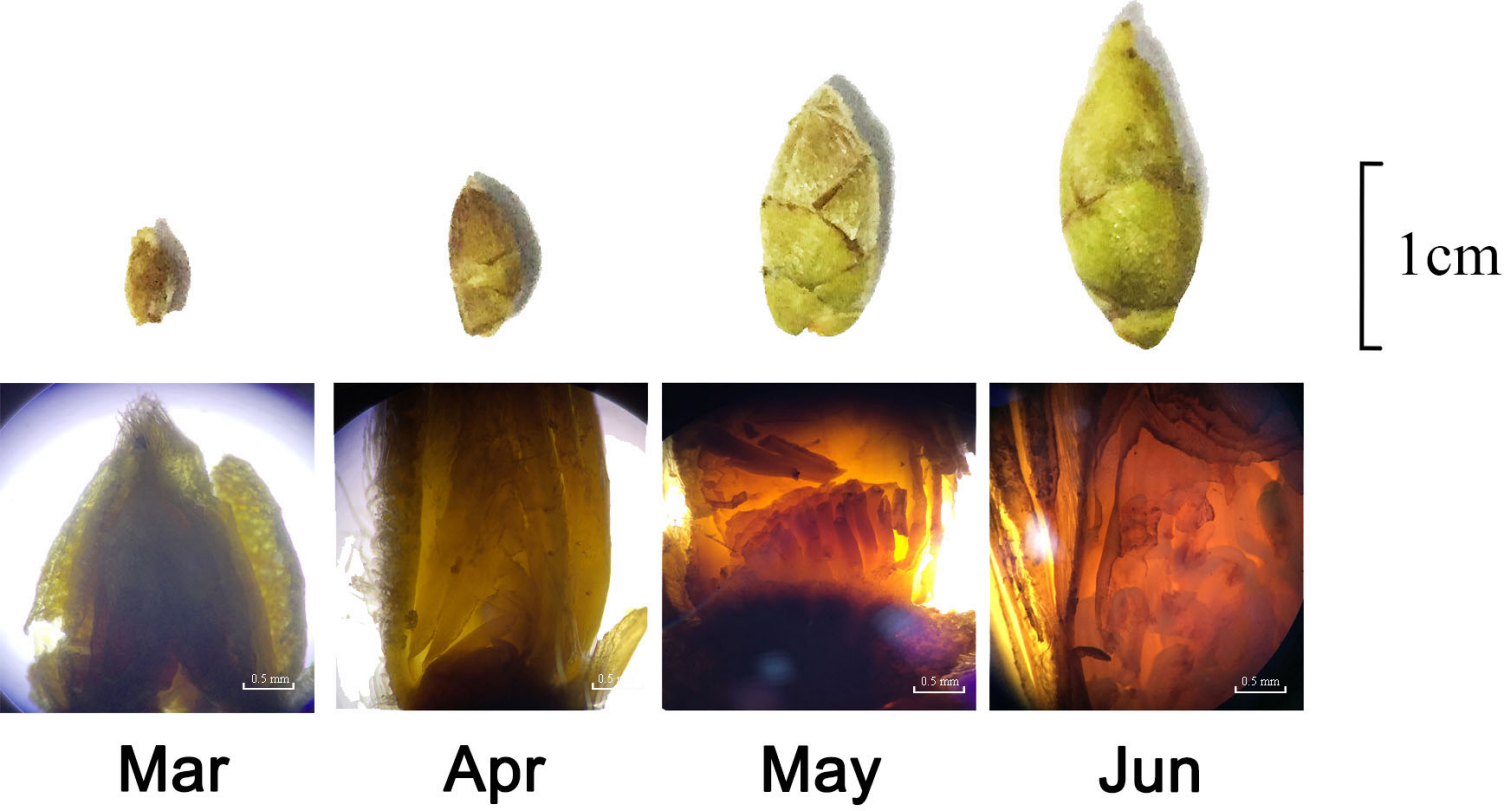
Observation on flower bud formation of *Camellia oleifera*.

The spring shoots of *C. oleifera* began to sprout in early March, and the growth of spring shoots slowed down or stopped in late March. The buds between the tips of the spring shoots or the axils of the leaves began to grow and differentiate; the cortex of the petiole and stem was slightly red, and the buds began to enter the physiological differentiation period to become flower buds. From the end of April to May, the buds entered the pre-differentiation period, where the growth point of the bud was slightly pointed. Meristem division was accelerated at the later growth stage; size was increased, and the tip was semicircular. Multiple growth cones are differentiated from the bud growth point (**Figure 3**). From May to June, the calyx primordium began to appear around the early growth point and then elongated and curled inward. The female and stamen primordium began to appear simultaneously, clearly distinguishing the flower bud from the leaf bud, and the flower bud was formed. Therefore, we divided the key period of flower bud formation (April–May) into four periods for subsequent experiments. From May to June, the calyx primordia began to appear around the early growing point; then the primordia elongated and curled inward, and the stamen and pistil began to appear simultaneously. Flower buds and leaf buds could be distinguished, and flower buds were formed. Accordingly, we selected the critical period for flower bud formation (April–May) and divided it into four stages (7, 14, 21, and 30 April) for subsequent experiments.

### 3.3 Analysis of hormone levels in different tissues during flower bud formation

Endogenous hormones in four tissues during the formation of *C. oleifera* flower buds are shown in **Table 1**. The distribution of the six endogenous hormones was markedly different among the four tissues. The IAA content was the highest in fruits, with an average value (average value of two cultivars) of 2,468.9 ng/g in the two cultivars during the stage of flower bud formation. The IAA content was low in branches and leaves (358.8 and 357.9 ng/g, respectively). The buds had the highest ABA content (3,535.7 ng/g), followed by the branches and fruits; the leaves had the lowest ABA content (110.5 ng/g). GA_3_ content was highest in shoots and fruits, followed by that in branches and leaves. The SA content was higher in branches and shoots (15,384.7ng/g and 11,772.5 ng/g, respectively) and lower in fruits and leaves (1/7 and 1/5 of that in branches, respectively). JA was mainly distributed in flower buds (512.6 ng/g), and the content of JA in fruits, branches, and leaves was similar (approximately 300 ng/g). The tZ content was one to two orders of magnitude lower in the tissues of *C. oleifera* than that of the other five endogenous hormones, with concentrations ranging from 10 to 30 ng/g, and the order of content from high to low was bud, leaf, branch, and fruit. Except for IAA and SA, the endogenous hormone levels were relatively high in oil tea buds and low in leaves.

**Table 1:** Endogenous hormone content in different tissues during flower bud differentiation of *C. oleifera* (Mean ± CV, ng/g).

Tissues	IAA	ABA	GA_3_	SA	JA	TZ
Bud	595.3 ± 19.0%	3,535.7 ± 27.4%	234.8 ± 22.8%	11,772.5 ± 20.9%	512.6 ± 37.1%	26.0 ± 22.3%
Fruit	2,468.9 ± 16.8%	782.2 ± 45.8%	244.5 ± 31.4%	3,628.3 ± 34.9%	311.1 ± 28.4%	10.6 ± 12.4%
Branch	358.8 ± 12.3%	1,804.6 ± 31.8%	137.9 ± 33.0%	15,384.7 ± 25.5%	296.9 ± 43.5%	13.7 ± 16.4%
Leaf	357.9 ± 22.0%	110.5 ± 39.9%	173.8 ± 33.9%	2,080.1 ± 53.5%	290.2 ± 46.4%	19.0 ± 46.5%

### 3.4 Analysis of endogenous hormone during flower bud formation

**Figure 4:**
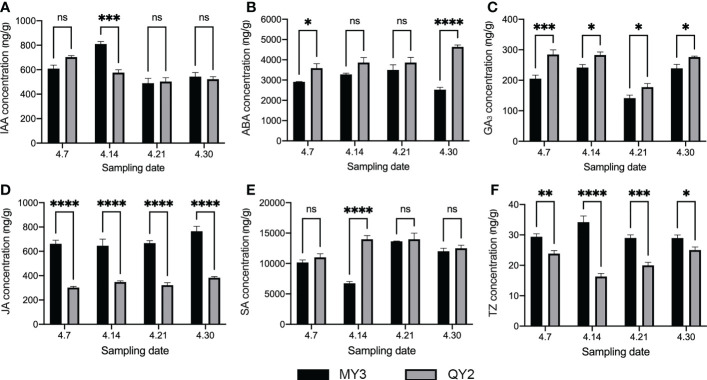
Endogenous hormones in buds during formation between two cultivars of *Camellia oleifera*. **A**: IAA; **B**: ABA; **C**: GA_3_; **D**: JA; **E**: SA; **F**:TZ. Black represents MY3, gray represents QY2. *: p<0.05, **: p<0.01, ***: p<0.001, ****: p<0.0001, ns: Not significant. All values represent the means of biological triplicates.

The endogenous hormone content of MY3 and QY2 in flower buds during flower bud formation is shown in **Figure 4**. The IAA content in the two cultivars was similar (**Figure 4A**), with a higher content in MY3 than in QY2 only on 14 April. No notable difference was found at other stages. The ABA content in QY2 was slightly higher than that in MY3 (**Figure 4B**), and the difference was most pronounced on 30 April. The ABA content in QY2 was above 3,700 ng/g during the entire flower bud formation stage, and that in MY3 fluctuated in the range of 2,400–3,700 ng/g. The GA_3_ content in QY2 was higher than that in MY3 at all four stages by approximately 15%–20%. The JA content was relatively stable during the four stages of flower bud formation; however, there was a considerable difference in JA content between the two cultivars. The JA content in MY3 buds was approximately twice that of QY2 buds. Like IAA content, SA only showed a significant difference between the two cultivars in the second stage (14 April). The SA content in QY2 was 14,340 ng/g, which was 112% higher than that in MY3. tZ showed the opposite trend for the two cultivars in the four stages. The tZ content in QY2 was 24.84 ng/g on 7 April, which then gradually decreased, reaching the lowest level of 16.31 ng/g on 14 April, followed by a gradual increase to a maximum level above 24 ng/g. The tZ content in MY3 first increased and then decreased, reaching the highest level of 34.55 ng/g on 14 April.

### 3.5 Transcriptomic changes involved in flower bud formation

In this study, 348.50 Gb data of RNA analysis were completed. The clean data of each bud sample was 16.51 Gb, and the percentage of Q30 base was above 92.94%. In total, 54,241 unigenes were produced after assembly, and 20,328 new genes were discovered, of which 11,463 were functionally annotated. Genes with differential expression patterns (FPKM fold change ≥2 or ≤0.5, *p <*0.05) between QY2 and MY3 cultivars were defined, and 6,319 differentially expressed genes (DEGs) were identified, including 3,704 in QY2 and 2,615 DEGs in MY3 (same cultivars in three periods), respectively. A total of 1,531 DEGs were identified during flower bud formation between two cultivars.

### 3.6 Functional annotation and classification of DEGs

**Figure 5:**
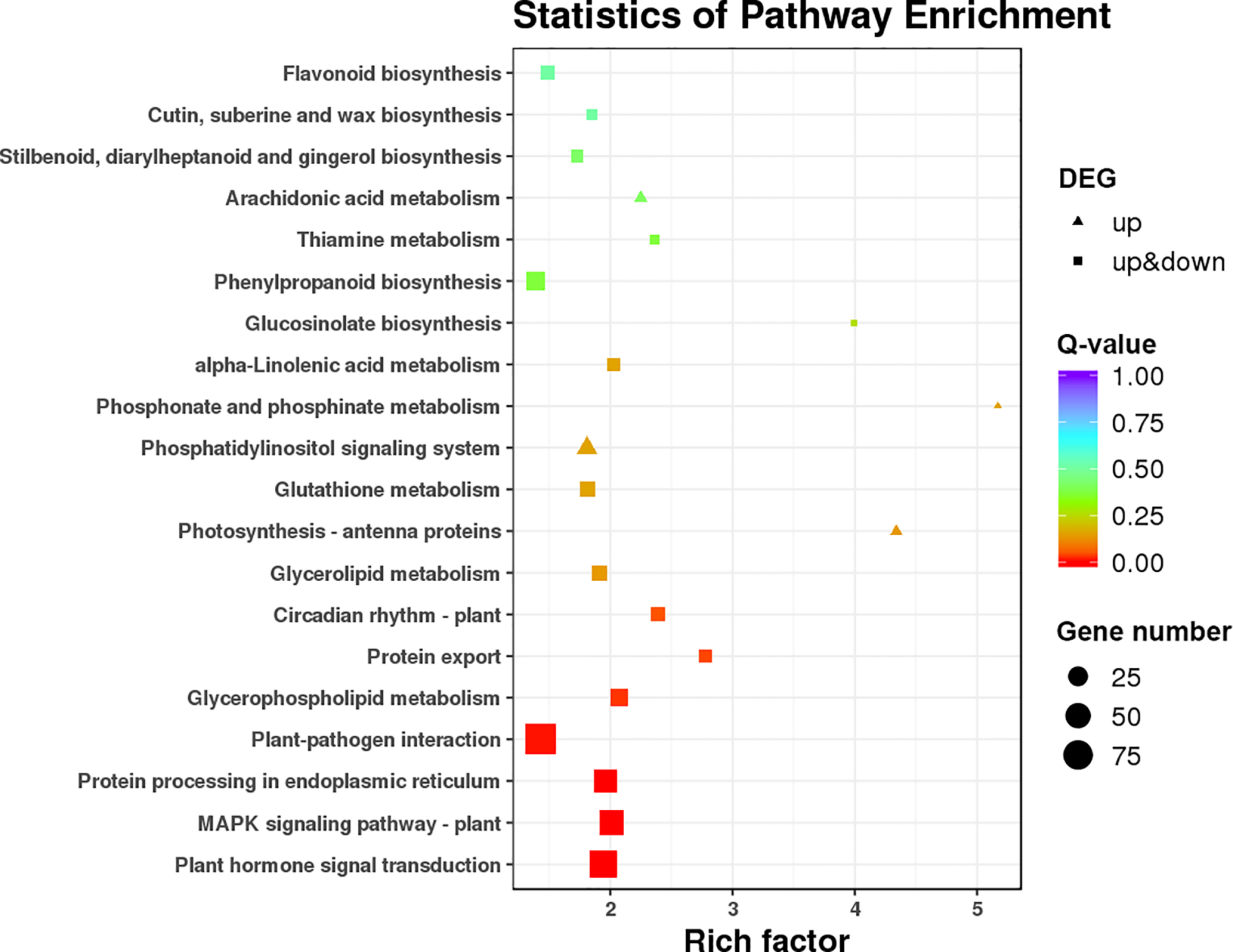
KEGG pathway enrichment for differently expressed genes.

KEGG pathway enrichment of DEGs was used to analyze whether DEGs appeared to be regulated by a certain pathway. The top 20 pathways with the lowest q values are shown in **Figure 5**. The DEGs were associated with various KEGG pathways involved in plant–pathogen interaction, the MAPK (mitogen-activated protein kinase) signaling pathway, plant hormone signal transduction, valine leucine and isoleucine degradation, and diterpenoid biosynthesis. To further reveal the reasons for alternate flower bud formation in QY2, plant hormone signal transduction, the circadian system, and flower meristem characteristic genes were further enriched and analyzed. Consequently, 41 key genes were successfully annotated and quantified, and the differences between two cultivars and three developmental stages were analyzed after classification.

### 3.7 Identification of genes related to plant hormone signal transduction in *C. oleifera*

**Figure 6:**
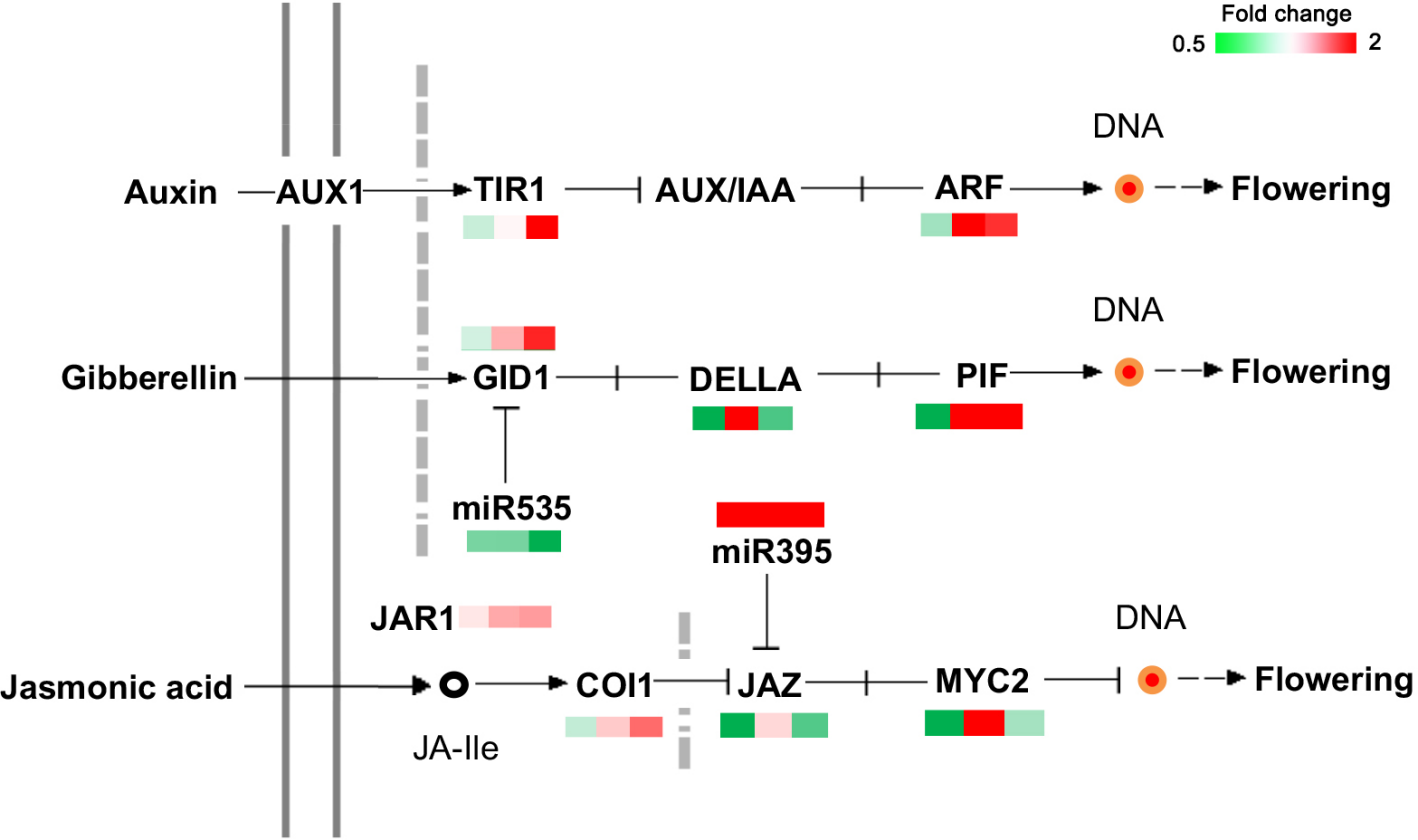
DEPs related to the plant hormone signal transduction in *Camellia oleifera*. (The pattern near each gene was representative of the ratio of their expression levels between MY3 and QY2. Left: MY3/QY2 at 4.14, MY3/QY2 at 4.21, right: MY3/QY2 at 4.30).

Based on the functional annotation and KEGG database, 20 genes involved in plant hormone signal transduction were identified (**Figure 6**). Among them, the IAA, GAs, and JA signaling pathways showed notable differences. *TIR1* (Transport inhibitor response 1), ARF8 (auxin response factor 8), *GID1c* (Gibberellin receptor GID1_C_), and *phytochrome-interacting factor 3* (PIF) were significantly upregulated in MY3 from 21 to 30 April. Inhibitors of flower bud formation, *DELLA* (DELLA protein GAI 1) and *MYC2* (transcription factor MYC2), were notably upregulated in MY3 on 21 April 2021 but downregulated at the first and third stages.

### 3.8 Identification of genes related to the circadian rhythm system in *C. oleifera*

**Figure 7:**
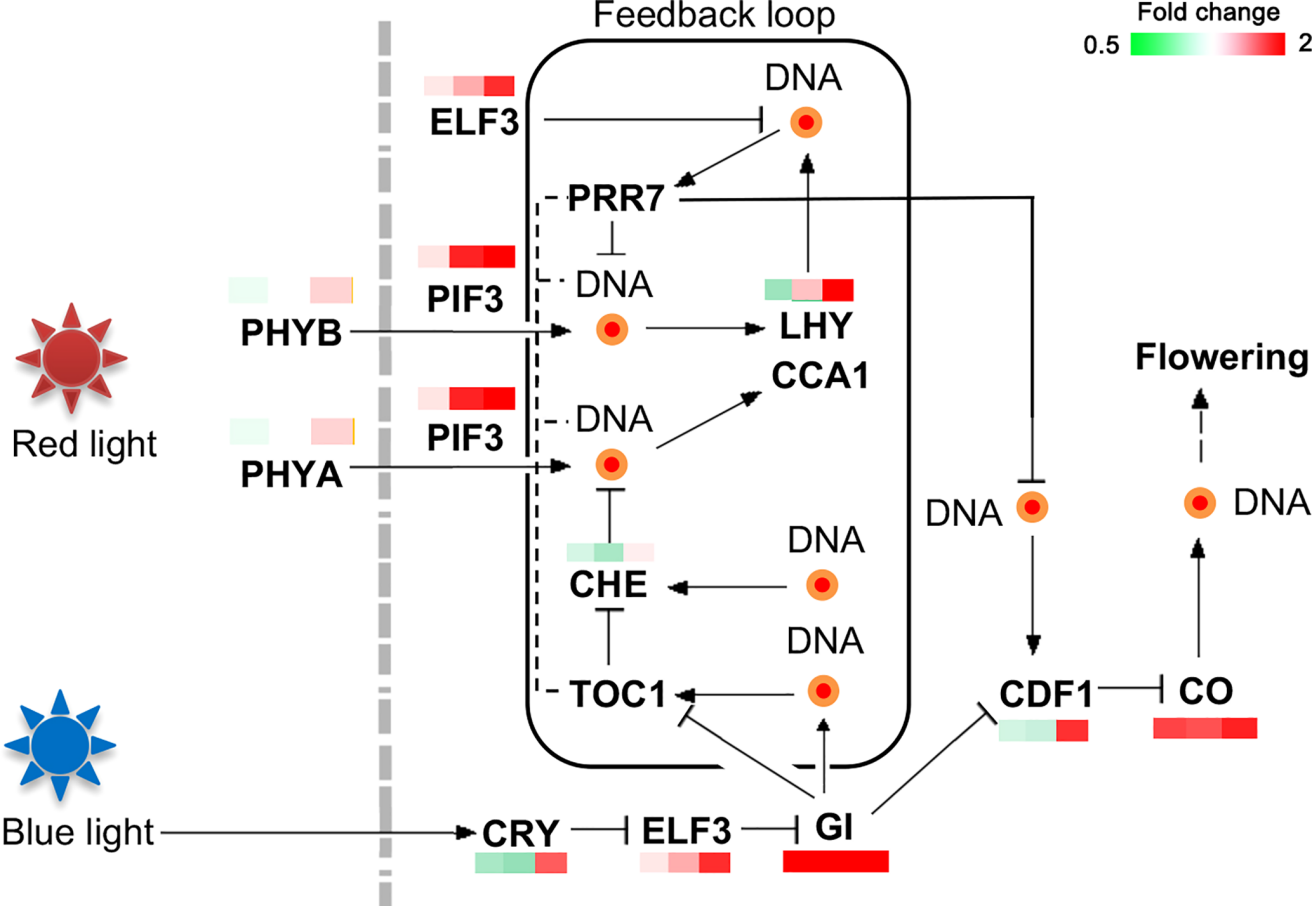
DEPs related to the circadian rhythm in *Camellia oleifera*. (The pattern near each gene was representative of the ratio of their expression levels between MY3 and QY2. Left: MY3/QY2 at 4.14, MY3/QY2 at 4.21, right: MY3/QY2 at 4.30).

The upregulated genes in the MY3 cultivar were *ELF3* (protein early flowering 3), *PIF3* (phytochrome-interacting factor 3), *GI*, *LHY* (MYB-related transcription factor LHY), *CDF1*, and *CO*. Most of these genes showed significant upregulation at the 21 and 30 April stages of flower bud formation (**Figure 7**). The light-sensitive signaling molecules *phytochrome A* (PHYA) and *phytochrome B* (PHYB) were not markedly different between the two cultivars. However, genes related to the red and blue light signal transduction pathways were enhanced in MY3.

### 3.9 Identification of genes related to flower bud formation in *C. oleifera*

**Figure 8:**
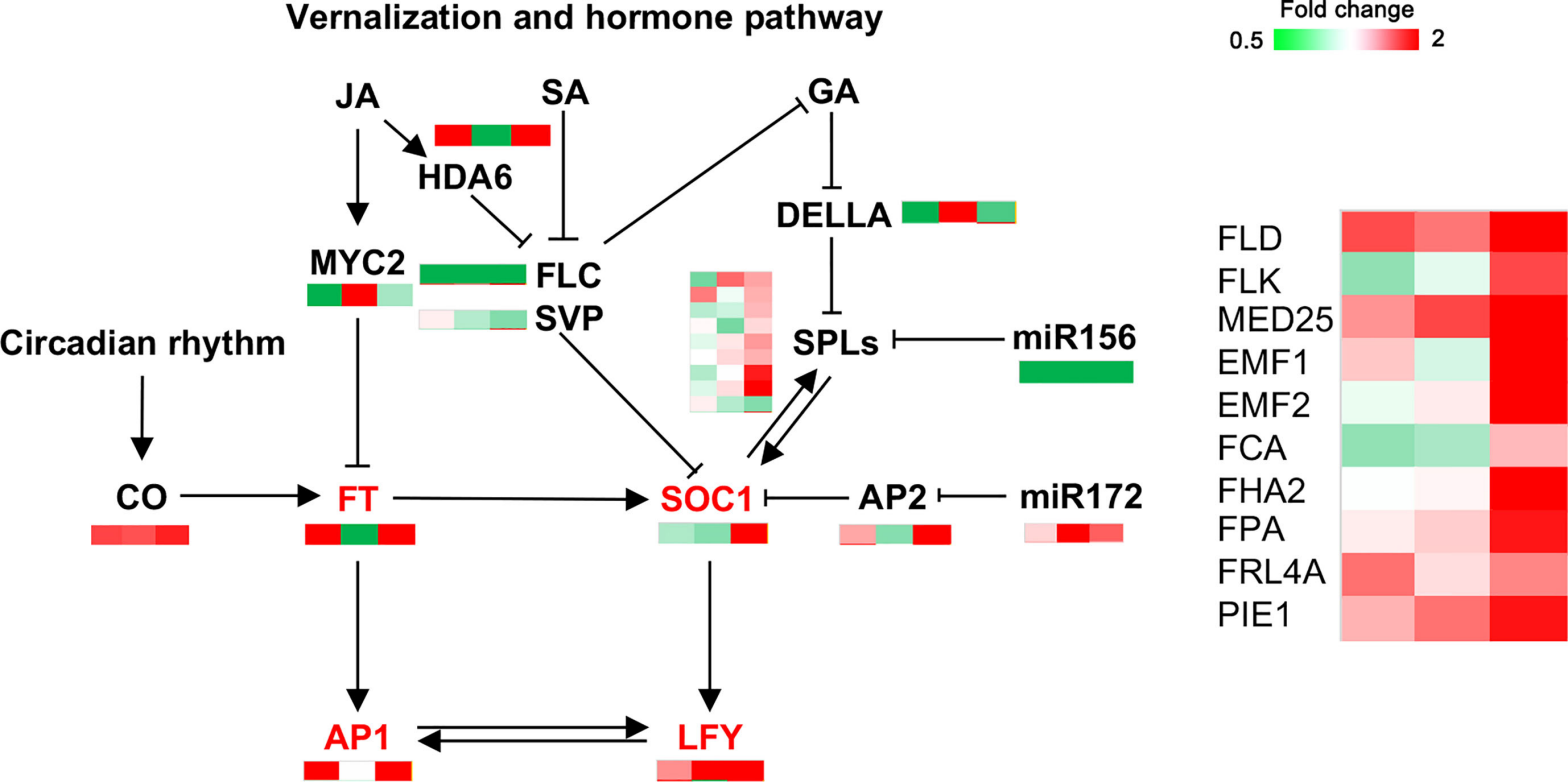
DEPs related to flower bud formation in *Camellia oleifera*. (The pattern near each gene was representative of the ratio of their expression levels between MY3 and QY2. Left: MY3/QY2 at 4.14, MY3/QY2 at 4.21, right: MY3/QY2 at 4.30).

Approximately 20 key genes involved in flower bud formation downstream of endogenous hormones (**Figure 8**, **Table S3**), vernalization, and circadian pathways were identified. Most flower bud-promoting genes, such as *FLD* (protein FLOWERING LOCUS D), *HDA6* (histone deacetylase 6), *FHA2* (FHA domain-containing protein FHA2), and *SPL3/6*, showed high expression in MY3. This was particularly evident at the 30 April stage. Similarly, floral meristem characteristic genes, including *SOC1*, *AP1* (floral homeotic protein APETALA 1), and *LFY*, were upregulated in MY3.

### 3.10 Identification of miRNAs related to flower bud formation in *C. oleifera*

**Figure 9:**
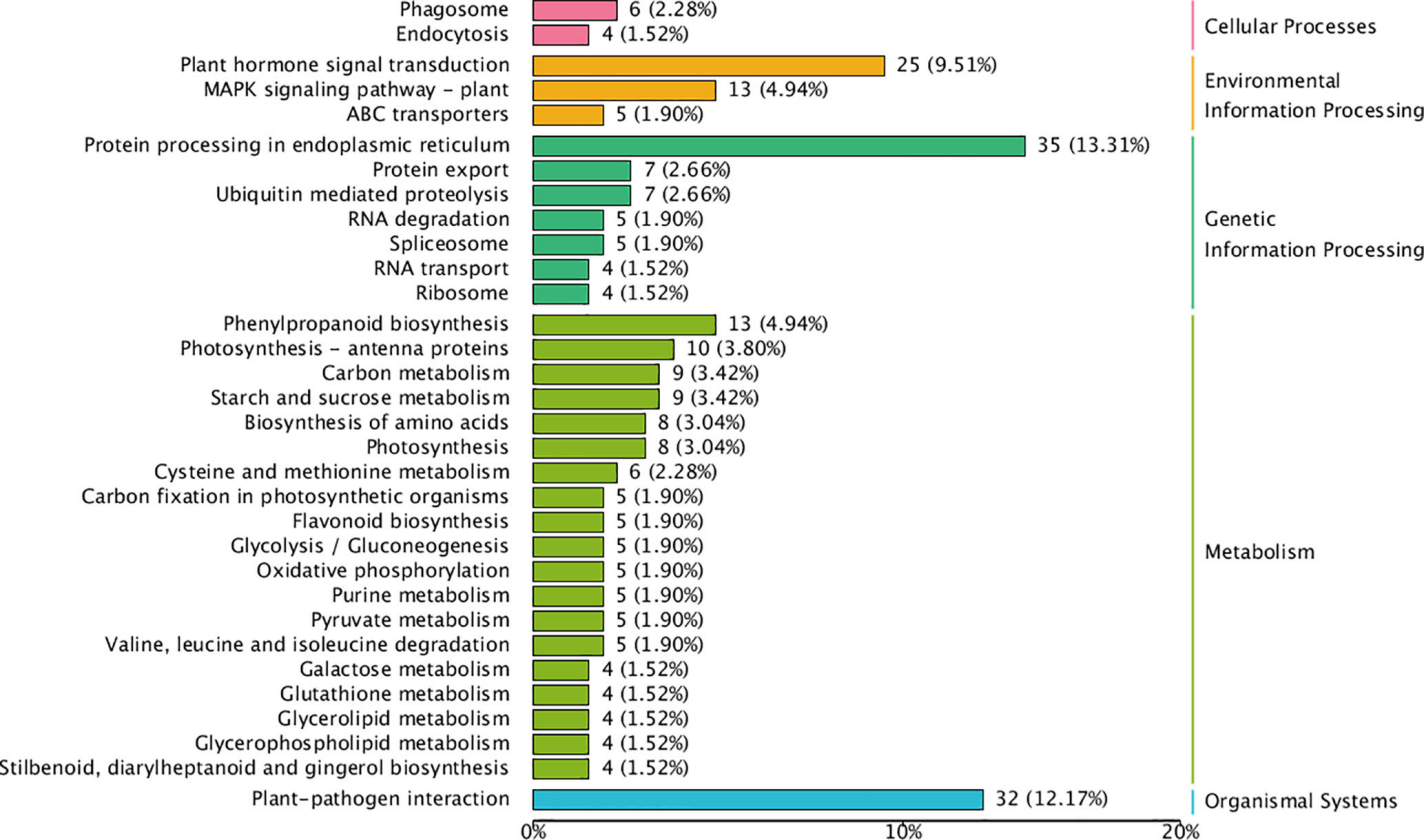
Pathway enrichment for target genes of significant difference miRNAs.

To comprehensively identify key miRNAs in the differentiating buds of stable and alternate cultivars of *C. oleifera*, high-throughput Illumina sequencing was performed for three different flower bud formation stages (14, 21, and 30 April), with three biological triplicates for each bud development stage of each cultivar. A total of 226.25 M clean reads were obtained, and no less than 9.45 M clean reads were obtained for each sample. More than 570 miRNAs were detected, including 209 known and 366 novel predicted miRNAs. miRNA expression levels in each sample were quantified. A total of 2,866 miRNA target genes were identified. The result of target gene enrichment analysis (fold change ≥1.5) is shown in **Figure 9**. The target genes of differentially expressed miRNA in cellular processes were predominantly related to phagosomes (2.28%) and endocytosis (1.52%). The target genes of DEMs in environmental information processing were mainly related to plant hormone signal transduction (9.51%), the MAPK signaling pathway (4.94%), and ABC transporters (1.91%). Regarding metabolism, 4.94% of the target genes were associated with phenylpropanoid biosynthesis, 3.8% with photosynthesis, and 3.42% with carbon metabolism. In addition, 12.71% of the genes enriched in organismal systems were related to plant–pathogen interactions. Four miRNAs were significantly different in the three stages of flower bud formation between the two cultivars, and their target genes were related to flower bud formation, namely miR535-*GID1c*, miR395-*JAZ* (jasmonate ZIM-domain), miR156-*SPLs*, and miR172-*AP2* (**Figures 6-8**).

### 3.11 Real-time PCR validation

Real-time PCR was performed on DEGs related to flower bud formation between QY2 and MY3 at the three developmental stages to validate the accuracy of the RNA-seq data. Approximately 10 DEGs, including *GID1c*, *JAZ*, *CO*, *MYC2*, *FT*, *SOC1*, *LFY*, *AP1*, *AP2*, *SPL3*, and four miRNAs, including miR535, miR395, miR156, and miR172, were identified. In both RNA-seq and qRT-PCR results, DEGs and miRNAs were consistently upregulated or downregulated (**Table 2**). Three selected miRNAs, miR535, miR395, and miR156, showed opposite expression trends relative to their target genes. Compared with QY2, the expression level of miR535 was slightly downregulated in the MY3 cultivar from 14 to 21 April, and significantly downregulated on 30 April. The target gene of miR535, *GID1c*, was upregulated on 30 April in MY3, and is obviously inhibited by miR535. The expression of miR395 in MY3 was more than two times higher than that in QY2 during the three stages of flower bud formation. The expression level of the target gene *JAZ* was reflected in the two periods of 14 and 30 April, which was more obvious in the results of qRT-PCR. The inhibitory effect on the target gene *JAZ* was reflected in the two periods of 14 and 30 April, which was more obvious in the results of qRT-PCR. Like the expression pattern of miR535, miR156 was downregulated in the MY3 cultivar from 14 to 21 April, and the target gene *SPL3* expression in MY3 showed upregulation in both the transcriptome and qPCR results on 30 April. Although miR172 was differentially expressed between the two cultivars, its target gene *AP2* did not show more than a two-fold difference in the process of flower bud formation.

**Table 2:** The comparison of mRNA-Seq/miRNA-seq and qRT-PCR analysis involved in flower bud formation in *C. oleifera*.

Protein name	Definition	mRNA-Seq/miRNA-seq data			qRT-PCR		
		MY3/QY2 April 14	MY3/QY2 April 21	MY3/QY2 April 30	MY3/QY2 April 14	MY3/QY2 April 21	MY3/QY2 April 30
GID1c	gibberellin receptor GID1c, alpha/beta-Hydrolases superfamily protein	0.91	1.16	2.13*	0.67	1.30	3.23*
JAZ	jasmonate ZIM domain-containing protein	0.41*	1.00	0.55	0.32*	0.89	0.42*
CO	zinc finger protein CONSTANS	1.86	1.84	1.93	1.19	2.14*	1.92
MYC2	transcription factor MYC2	0.27*	2.65	0.82	0.19*	2.12*	0.79
FT	FLOWERING LOCUS T	4.31*	0.15*	5.53*	6.52 *	0.20*	10.28*
SOC1	Suppressor of overexpression of CO 1	0.84	0.76	2.43*	1.21	1.19	4.53*
LFY	LEAFY	1.22	1.92*	2.61*	1.01	2.61*	3.17*
AP1	APETALA 1	2.18*	1.01	2.81*	3.13*	1.30	4.92*
AP2	APETALA 2	1.18	0.76	1.86	0.72	0.52	1.13
SPL3	SQUAMOSA BINDING FACTOR-LIKE 3	0.84	1.01	2.44*	1.12	0.83	3.33*
miRNA	miR535	0.72	0.73	0.42*	0.95	0.61	0.32*
miRNA	miR395	2.18*	2.31*	2.53*	2.38*	4.32*	3.16*
miRNA	miR156	0.43*	0.38*	0.45*	0.31*	0.49*	0.36*
miRNA	miR172	1.21	2.47*	1.56	1.51	3.43*	1.42

qRT-PCR analysis of cDNA isolated from the buds at 14, 21. and 30 April. The actin gene was used as an internal standard. Values represent the means of three biological triplicates. *Indicates a significant difference at 0.05 level.

## 4 Discussion

### 4.1 Hormones and hormone signaling pathways involved in flower bud formation

Flower bud formation is an adaptive response of plants to environmental changes under a complex floral regulatory network formed by a variety of exogenous and endogenous signals (Zou et al., 2020). Plant hormones (IAA, GA, JA, and tZ), the most important endogenous signals, play an important regulatory role in plant flower bud formation (Larsson et al., 2013; Ohri et al., 2015). GA_3_, JA, and tZ contents were notably different in the two cultivars during all stages of flower bud formation, thereby suggesting that they might play an important role in regulating the bud formation of *C. oleifera*. GA signal transduction is mainly achieved by GID1 (GA insensitive dwarf 1), which mediates degradation of DELLA proteins (Fukazawa et al., 2021) (**Figure 6**). DELLA proteins can bind to various transcription factors to simultaneously inhibit CO protein function and *FT* transcriptional activation (Sun, 2011). At present, the effect of GA on flower bud formation is still controversial, but studies in the past two decades have revealed that GA is necessary for flower bud formation. The GA-deficient mutant *ga1-3*, which is deficient in the *GA1* gene encoding the very first enzyme involved in GA biosynthesis, fails to flower under short days (Wilson et al., 1992). Porri found that GA could also up-regulate the expression levels of *FT* and the twin sister of FT (TSF) and promote the formation of flower buds under long photoperiods (Porri et al., 2012). Therefore, high concentrations of GA_3_ or high efficiency of GA_3_ signal transduction (low concentrations of DELLA) contribute to flower bud formation in plants (Davière and Achard, 2016) (**Figure 8**). But another view is that GA can promote vegetative growth and inhibit reproductive growth. Gibberellin inhibitor uniconazole (6.9 μM) increased the number of adventitious shoots formed by as much as twofold but decreased shoot length by about 50% (Sankhla et al., 1994). With the application of GA inhibitors, significant opposite cytological and morphological changes were observed in treated terminal buds, which led to a reduced flowering rate under gibberellin and an increased flowering rate under paclobutrazol (Lee and Lee, 2010; Fan et al., 2018). The content of GA_3_ in MY3 buds was significantly lower than that of QY2 buds during the whole flower bud formation process, and the content of both cultivars decreased at the stage of 21 April and then increased, indicating that the promotion of *C. oleifera* bud formation might require the fluctuation of endogenous GA levels in the key period and that low concentrations of GA_3_ were conducive to bud formation. This was consistent with the result that exogenous application of paclobutrazol filing promoted the production of more flower buds in woody oil species (Seesangboon et al., 2018). In addition, the expression of *DELLA*, the key regulatory factor in the GA signaling pathway, also showed such fluctuations. Correspondingly, the key genes regulating flower bud formation, such as *SPL3*, *FLD*, and *SOC1*, were significantly expressed in MY3 after April 21 (**Figure 8**). These results indicated that 21–30 April was the key period for regulating *C. oleifera* bud formation at the hormone level.

IAA not only affects the elongation, differentiation, and other physiological processes of plant cells (Dinesh et al., 2016), but also participates in plant floral regulation. Mai et al. (2011) found that the IAA-deficient mutant *axr2* (auxin-resistant 2) delayed flower formation in short-day light. Exogenous application of different concentrations of IAA can affect the normal development of flowers (Jaligot et al., 2000). IAA signaling pathway analysis showed that MY3 enhances flower bud formation through increasing the expression of *TIR1* and *ARF* during 21–30 April and has an obvious synergistic effect with the GA signaling pathway. CTK not only promotes stem elongation but also plays a role in flower bud formation. Corbesier found a rapid increase in endogenous CTK content after floral stimulation, confirming that CTK plays the same role as IAA in promoting flower bud formation (Corbesier et al., 2003). Exogenous application of CTK induces floral transition at the cellular level in the vegetative growth phase under short-day light (Corbesier et al., 2003). MY3, with more flower buds, formed, and the tZ content was higher than QY2, indicating that the increase in tZ content in *C. oleifera* promoted the flowering transition. JA and its derivatives are known as lipid phytohormones. The signaling pathway of JA and its response mechanism to stress have been extensively analyzed (Berger, 2002). JA is an essential hormone for the normal development of flowers in plants, and filament elongation, pollen maturation, and anther dehiscence were affected in the JA-deficient mutant *Arabidopsis* (Zhang et al., 2014a). But the role of JA in regulating the induction of flower bud formation has not been well studied. A few studies have shown that JA plays an inhibitory role in bud formation in *Arabidopsis*, which inhibits *FT* expression and delays flowering *via* the JAZ signaling pathway (Zhai et al., 2015; Wang et al., 2017). Many pieces of evidence showed that the JA signaling pathway had a synergistic effect on GA and DELLA proteins directly or indirectly regulated the function of MYC2. This could be confirmed by the same expression pattern of DELLA and JAZ in the two *C. oleifera* cultivars. DELLAs usually compete with MYC2 to bind JAZ (Hou et al., 2010). JA in MY3 buds was higher than QY2. JA would form a complex with JAZ to degrade it. The decrease in JAZ content reduced its occupation of DELLAs, thereby enhancing the role of the GA signaling pathway and promoting flower bud formation.

Previous studies on fruit trees had shown that fruit could affect flower bud formation (Munoz-Fambuena et al., 2011; Martinez-Alcantara et al., 2015). In the harvest year, the synthesis of gibberellin from seed embryos also increases significantly (Milyaev et al., 2022). Therefore, it could be inferred that this phenomenon would be more obvious in *C. oleifera*. In this study, we also found that higher GA_3_ concentrations may affect flower bud formation, which is consistent with the results of previous studies. However, the results of hormone content determination in different tissues exclude the influence of fruit hormone on flower bud formation, which could be promoted by exogenous hormone or a hormone inhibitor without yield reduction.

### 4.2 Circadian rhythm involved in flower bud formation

In the photoperiodic pathway, the time and intensity of light are sensed by phytochromes, and the corresponding response is generated to form a circadian rhythm. Changes in the length of the day and night could disrupt the balance and lead to the expression of *CO*, *FT*, and *SOC1*, which induce flower bud formation or inhibition (Alabadí et al., 2002). Previous studies have shown that photoperiod regulation is superior to the GA signaling pathway, and the importance of the GA signaling pathway has only been highlighted when photoperiod regulation does not play a dominant role (Campos-Rivero et al., 2017). The phytochrome genes (*PHYA* and *PHYB*) did not show notable differences between the two cultivars. It is most likely not regulated at the transcriptional level, which is consistent with the findings in poplars (Shim et al., 2014). The circadian rhythm of *C. oleifera* promotes flower bud formation mainly by upregulating the expression of the phytochrome interaction factor PIF3 (PHYTOCHROME-INTERACTING FACTOR 3). PIF is inhibited by DELLA in the GA signaling pathway (Li et al., 2016), and downregulation of DELLA in the middle and late stages of MY3 flower bud formation could further relieve the repression of PIF (Richter et al., 2010). PIF induces flower bud formation by directly or indirectly regulating the expression of *GI*, *CO*, and *FT* in MY3 (Galvāo et al., 2019).

### 4.3 Signal transduction pathways involved in flower bud formation

The transition from shoot apex meristem to inflorescence meristem generation in plants is mediated by a complex gene regulatory network (**Figure 8**) (Freytes et al., 2021). However, the vernalization, GA, photoperiod, and autonomous pathways all converge on the two major flower bud formation integration proteins, FT and SOC1, through their respective flowering signals (Nakano et al., 2011). The integrated genes further activate the floral meristem signature genes, *LFY* and *AP1*, ultimately generating the inflorescence meristem (Périlleux et al., 2019). In this study, the changes in *FT* and *SOC1* expression showed that *FT* and *SOC1* in buds were regulated by the hormone pathway, and *FT* expression was markedly inhibited by MYC2 in the three stages of flower bud formation. These results were consistent with research on *Castanea mollisima* (**Figure 8**) (Cheng et al., 2022). FT can also be generated in the leaves and moved to the SAM (shoot apical meristem). In SAM, FT does not directly bind to DNA (Lei et al., 2017), but rather acts as a transcriptional co-regulator of AP1 by interacting with the transcription factor FD (bZIP transcription factor) (Taoka et al., 2011). miR156 and SPL play opposite roles in regulating flower bud formation. Overexpression of miR156 delays flowering time, whereas SPL promotes flowering under both long- and short-day conditions (Wang et al., 2009). The comparison of MY3 and QY2 showed that the expression of *SOC1* positively correlated with *SPL6* and *SPL3*, thereby suggesting that SPL6 and SPL3 in the SPL family play a role in regulating SOC1 during bud formation in *C. oleifera* (Jung et al., 2012). *FLD* and *FLK* (FLOWERING LOCUS WITH KH DOMAINS) encode RNA-binding proteins with K homology motifs and regulate flowering time *via* FLC (Lim et al., 2004). They were highly expressed in MY3 (30 April) and promoted flower bud formation in MY3 plants. Like the expression patterns of *FLD* and *FLK*, other genes related to flower bud formation were significantly different between the two cultivars on 30 April. Combined with hormonal and signal transduction pathways, the regulation of *C. oleifera* flower bud formation mainly occurred after 21 April at both the hormone and transcriptome levels.

### 4.4 miRNA–mRNA modules involved in flower bud formation

Many studies have demonstrated that miRNAs regulate flower bud formation by regulating the expression of their target genes, such as miR172-*AP2*, miR159-*MYB*, miR319-*TCP*, miR167-*ARF6/8*, miR156-*SPLs*, miR167, miR169, miR172, miR319, miR390, and miR399 (Hong and Jackson, 2015). In this study, miR156-*SPL3* and miR172-*AP2* modules showed a significant difference between two *C.* cultivars during flower bud formation. The low expression of miR156 in MY3 relieved the inhibitory effect on SPL3 in the middle and late stages of flower bud formation. SPL3 has been shown to bind to the promoters of the floral meristem recognition genes *LFY*, *FUL* (FRUITFULL), and *SOC1* to promote flower bud formation (Yamaguchi et al., 2009). The high expression of its downstream genes *SOC1* and *LFY* in MY3 confirmed the promoting effect of the miR156-*SPL3* module on flower bud formation in *C. oleifera*. Studies have shown that the miR172-*AP2* module is involved in the control of flower development, and misexpression of rAP2 from heterologous promoters showed that AP2 acts on meristem size and the rate of flower production. However, miR172 and its target gene *AP2* and the downstream *SOC1* gene did not show a significant regulatory relationship in MY3 and QY2, which indicated that the miR172-*AP2* module did not play a major regulatory role in the formation of *C. oleifera* flower buds.

MiR535-*GID1c* and miR395-*JAZ* regulatory modules related to flower bud formation in hormone signaling pathways were first found in this study. Several studies in recent years showed that miR535 had an important regulatory role in the growth of peaches, rice, and potatoes (Zhang et al., 2014b; Shi et al., 2017). Overexpression of miR535 reduced plant height by shortening the first or second internodes of rice (Sun et al., 2019); the panicles were more numerous and shorter. It was speculated that miR535 might inhibit the expression of *SPL7/12/16* (Sun et al., 2019). In the study, bioinformatics prediction results showed that miR535 targeted the receptor protein *GID1c* in the GA signaling pathway. The expression patterns of *GID1c* and miR535 were opposite in two *C. oleifera* cultivars, indicating that there may be a regulatory relationship between them. In MY3, low expression of miR535 during the critical period of flower bud formation (21–30 April) relieved its inhibition of *GID1c*. The increased GID1c content might promote the transduction efficiency of the GA signaling pathway in MY3 and promote the formation of flower buds. miR395 has been reported to be related to plant immunity and plant nutrient stress. There was evidence that miR395 targets and regulates the expression of *OsAPS1*, *OsSULTR2;1*, and *OsSULTR2;2*, which function in sulfate assimilation and translocation, promote sulfate accumulation, resulting in broad-spectrum resistance to bacterial pathogens in miR395 overexpressed plants (Yang et al., 2022). In the study, bioinformatics prediction results showed that miR395 targeted JAZ in the JA signaling pathway. As a negative regulator in the JA signaling pathway, JAZ is degraded by 26S protease to release the positive regulatory transcription factor MYC2 in the JA signaling pathway, opening the transcription of early JA response genes (Liu et al., 2021). According to the results of the transcriptome and qRT-PCR, miR395 had a certain inhibitory effect on the *JAZ* gene. The high expression of miR395 in MY3 inhibited the expression of *JAZ* and weakened the inhibitory effect of JAZ on the JA signaling pathway. Combined with the result that the content of JA in MY3 was more than twice as high as that of QY2, it indicated that high concentrations of JA and the removal of JA signaling pathway inhibition had a significant promoting effect on the formation of flower buds.

## Data availability statement

The datasets presented in this study can be found in online repositories. The names of the repository/repositories and accession number(s) can be found below: https://www.ncbi.nlm.nih.gov/, PRJNA905846.

## Author contributions

CR designed the research. WD performed the experiments, analyzed the data, and wrote the manuscript. All authors contributed to the article and approved the submitted version.
