# Supplementary material for: Co-regulatory effects of hormone and mRNA–miRNA module on flower bud formation of Camellia oleifera
Source: Front Plant Sci. 2023 Mar 17;14:1109603. doi: 10.3389/fpls.2023.1109603 (PMC10064061; doi:10.3389/fpls.2023.1109603)
Supplement: Supplementary file 1 [file Table_1.docx]

Supplementary Material

# Supplementary Tables

**Table S1.** LC-MS/MS parameters and quantitative information of endogenous hormones.

| **Hormones** | **Scan mode** | **Q1 Mass (Da)** | **Q3 Mass (Da)** | **DP (Volts)** | **CE (Volts)** | **Retention Time (min)** |
| --- | --- | --- | --- | --- | --- | --- |
| IAA | ESI (+) | 176.1 | 130.2*/102.9 | 35 | 20 | 1.78 |
| ABA | ESI (-) | 263.1 | 218.9/153.1* | -41 | -17 | 1.26 |
| GA_3_ | ESI (-) | 345.1 | 239.2* | -45 | -40 | 0.85 |
| JA | ESI (-) | 209.2 | 58.9* | -58 | -50 | 1.66 |
| SA | ESI (-) | 136.8 | 92.8* | -25 | -40 | 0.68 |
| TZ | ESI (+) | 220.2 | 136.3*/118.9 | 54 | 22 | 0.81 |

**Supplementary Table 2.** Primer sequences used in the PCR experiments.

| **Enzyme Symbol** | **Sequence (5'-3')** |
| --- | --- |
| GID1c | F: ATTGTGCTCCAGTGGGTCTG |
|  | R: CTACATGCTGAACCGCTTGC |
| JAZ | F: CCGTGGAAGAGTCCCTCAAC |
|  | R: GCCGGTTTGACTCTACTGCT |
| CO | F: GATACGGCGTCGTTCCTTCT |
|  | R: CAAAATCGACGGGCAGCAAT |
| MYC2 | F: ATCCCGGTTTTCAGGTCCAC |
|  | R: AGCTGCAGGGTGGTTCTTTT |
| FT | F: CCGAGTGGTTCTTCCTCGTC |
|  | R: CGGAATCGACCATGAACCCA |
| SOC1 | F: ATTGGTTGGGACGCGATGAT |
|  | R: TTCACCGTGGCTTGTTGGAT |
| LFY | F: TGGTCGATTCCGGCAGAAAA |
|  | R: GGTCTGAATCTCCGCCACAA |
| AP1 | F: GAAGACAAAGGCAAGCGCAA |
|  | R: TTTTCTGCCGGAATCGACCA |
| SPL3 | F: GACCACTCTGACCTCGAAGC |
|  | R: TTCGAATCATCGCGTCCCAA |
| MIR535 | F:TTATCGTCCAAAATTGGCG |
|  | R:CAGTGCAGGGTCCGAGGTAT |
| MIR395 | F:CCGATCCATCGTCGTCCGTG |
|  | R:CAGTGCAGGGTCCGAGGTAT |
| MIR156 | F:AATTGAGTCCTTAAAAAGAGA |
|  | R:CAGTGCAGGGTCCGAGGTAT |
| MIR172 | F:GGTGAAATCGAGCGGCATGTTC |
|  | R:CAGTGCAGGGTCCGAGGTAT |
| Actin | F: GAAACTACGGTTGCGGATAGAG |
|  | R: CTCCGGTGCATCCTTCATAAT |

| **Accession Number** | **Gene name** | **Definition** | **FPKM** | | | | | |
| --- | --- | --- | --- | --- | --- | --- | --- | --- |
|  |  |  | **MY3**  **April 14** | **MY3**  **April 21** | **MY3**  **April 30** | **QY2**  **April 14** | **QY2**  **April 21** | **QY2**  **April 30** |
| TEA011986 | HDA6 | Histone deacetylase 6 | 31.41 | 30.93 | 33.77 | 31.91 | 29.55 | 22.68 |
| TEA006583 | FLC | Flowering locus C | 9.60 | 15.68 | 15.42 | 3.20 | 3.09 | 3.54 |
| TEA008716 | SVP | MADS-box protein SVP | 14.93 | 14.47 | 11.57 | 14.36 | 17.04 | 15.29 |
| TEA014556 | SOC1 | Suppressor of overexpression of co 1 | 5.47 | 3.81 | 5.77 | 6.53 | 4.98 | 2.37 |
| TEA015714 | FT | Flowering Locus T | 5.52 | 1.11 | 14.85 | 1.28 | 7.33 | 2.69 |
| Camellia34015 | SP1 | APETALA 1 | 2.12 | 1.46 | 1.20 | 1.20 | 1.44 | 0.66 |
| TEA003201 | LFY | LEAFY | 2.06 | 3.43 | 5.75 | 1.69 | 2.26 | 3.58 |
| TEA033058 | AP2 | APETALA 2 | 8.06 | 5.89 | 8.24 | 6.85 | 7.72 | 4.97 |
| TEA001755 | SPL3 | Squamosa promoter-binding-like protein 3 | 22.29 | 22.40 | 17.15 | 26.42 | 22.11 | 6.10 |
| TEA030618 | SPL6 | Squamosa promoter-binding-like protein 6 | 16.65 | 19.89 | 26.06 | 18.02 | 18.52 | 17.24 |
| Camellia6970 | FLD | Flowering Locus D | 7.16 | 5.89 | 6.62 | 5.31 | 4.64 | 4.41 |
| TEA022841 | FLK | Flowering Locus K | 42.90 | 42.35 | 37.17 | 54.86 | 44.96 | 27.43 |
| TEA003688 | MED25 | Mediator of RNA polymerase II transcription subunit 25 | 39.26 | 43.14 | 40.34 | 32.33 | 31.72 | 24.97 |
| TEA024300 | EMF1 | Embryonic Flower 1 | 5.81 | 5.54 | 5.53 | 5.21 | 6.05 | 3.31 |
| TEA011052 | EMF2 | Embryonic Flower 2 | 42.10 | 42.65 | 38.64 | 44.15 | 40.66 | 24.91 |
| Camellia102028 | FCA | Flowering time control protein | 11.03 | 9.89 | 11.72 | 14.09 | 11.91 | 10.27 |
| TEA024884 | FHA2 | FHA domain-containing protein FHA2 | 11.66 | 11.10 | 12.01 | 11.84 | 10.77 | 7.57 |
| TEA013644 | FPA | Flowering time control protein FPA | 27.63 | 27.53 | 36.51 | 26.42 | 24.90 | 25.13 |
| TEA005263 | FRL4A | FRIGIDA-like protein 4a | 289.70 | 265.28 | 316.76 | 226.49 | 247.14 | 255.37 |
| Camellia11071 | PIE1 | Photoperiod-Independent Early Flowering 1 | 6.25 | 5.92 | 6.33 | 5.42 | 4.64 | 4.34 |

**Supplementary Table 3. Information and expression of 20 genes related to flower bud formation.**
